# The relation between obesity and depressed mood in a multi-ethnic population. The HELIUS study

**DOI:** 10.1007/s00127-018-1512-3

**Published:** 2018-04-11

**Authors:** Deborah Gibson-Smith, Mariska Bot, Marieke Snijder, Mary Nicolaou, Eske M. Derks, Karien Stronks, Ingeborg A. Brouwer, Marjolein Visser, Brenda W. J. H. Penninx

**Affiliations:** 1 Department of Psychiatry, Amsterdam Public Health Research Institute, VU University Medical Center, Oudenaller 1, 1081 HJ Amsterdam, The Netherlands; 2 Department of Public Health, Amsterdam Public Health research institute, Academic Medical Center, Amsterdam, The Netherlands; 3 Department of Clinical Epidemiology, Biostatistics and Bioinformatics, Amsterdam Public Health research institute, Academic Medical Center, Amsterdam, The Netherlands; 4 Department of Psychiatry, Academic Medical Center, Amsterdam, The Netherlands; 5 QIMR Berghofer, Translational Neurogenomics group, Brisbane, Australia; 6 Department of Health Sciences, Faculty of Earth and Life Sciences and Amsterdam Public Health research institute, Vrije Universiteit Amsterdam, Amsterdam, The Netherlands; 7 Department of Internal Medicine, Nutrition and Dietetics, VU University Medical Center, Amsterdam, The Netherlands

**Keywords:** Depressed mood, Obesity, Overweight, Ethnicity, HELIUS study

## Abstract

**Purpose:**

To examine the association between obesity and depressed mood in a large multi-ethnic population and check for consistency in this association across six ethnic groups.

**Methods:**

Data of 21,030 persons (18–70 years) were sourced from the HELIUS study. Cross-sectional relationships between obesity measures [body mass index (kg/m^2^) and waist circumference (cm)] and depressed mood (PHQ-9 score ≥ 10) were analysed. Consistency of associations was investigated across ethnic groups by interaction terms (ethnicity*obesity measures) in basic (age, sex, education) and fully (health behaviours and somatic health) adjusted models.

**Results:**

Obesity was prevalent in all ethnic groups, but varied substantially. After sociodemographic adjustment, obesity measures were associated with increased odds of depressed mood but this was inconsistent across ethnic groups. Obesity (BMI ≥ 30 or highest waist circumference quartile) was strongly and significantly associated with depressed mood in the Dutch [Odds Ratio (OR) = 1.72; 95% Confidence intervals (CI) 1.24–2.40, and OR = 1.86; 95% CI 1.38–2.50], respectively, and African Surinamese (OR = 1.60; 95% CI 1.29–1.98 and OR = 1.59; 95% CI 1.27–2.00, respectively) but had a weaker, non-significant association in other ethnic groups (South-Asian Surinamese, Ghanaian, Moroccan, Turkish groups). Adjustment for health behaviours and somatic health had limited effect on this pattern.

**Conclusion:**

Obesity was associated with a higher risk of depressed mood. However, ethnic differences were found: the obesity-depressed mood association was strong in the Dutch and African Surinamese populations, but not in other ethnic groups. Future studies should explore whether differential normative values or pathophysiology across ethnic groups explain why the obesity-depression association is inconsistent across ethnic groups.

**Electronic supplementary material:**

The online version of this article (10.1007/s00127-018-1512-3) contains supplementary material, which is available to authorized users.

## Introduction

Obesity is a growing public health problem [1] that has been associated with many short-term effects such as social stigmatisation [2] and joint pain [3], and longterm negative health outcomes such as diabetes [4], hypertension [5] and some cancers [6]. Cross-sectional reviews and meta-analyses have shown that obesity and depression are associated [7]. Furthermore, longitudinal studies analysing the temporal trends between obesity and the development of depression have demonstrated that a higher BMI, and in particular obesity (BMI ≥ 30 kg/m^2^), increases the risk of developing depression and vice versa [8]. Depression is an important public health concern [9] as depression is the leading cause of years lived with disability [10].

Several studies from the United States suggest that the association between obesity and depression may differ between ethnic groups [11–17]. Most studies observed a stronger association among White and Hispanic Americans as compared to Blacks [11–16]. One longitudinal study, however, found that obesity had a stronger association with depression for African Americans, as opposed to White Americans [17]. In addition, the literature is not entirely unanimous with several studies finding no differences across ethnic groups [18, 19]. Moreover, existing literature is limited to the study of White Americans, Afro-Caribbean Americans and Hispanics, thereby omitting minority groups more commonly found in Europe. For Example in the Netherlands, apart from the ~ 90% Dutch and other Western origins, the largest ethnic groups comprise 2.3% Turkish, 2.3% Moroccan, 2.1% Surinamese, 0.9% Antilleans and 4.5% other [20].

Diverse mechanisms explaining ethnic differences in the causal link between obesity and depression have been proposed. First, differences may be due to the fact that obesity is more prevalent and, therefore, more normative in certain cultural groups, thus having a smaller impact on mental health. Contributing attributes here could be psychosocial factors such as greater body dissatisfaction or increased weight discrimination. Second, health behaviours, such as alcohol consumption, smoking behaviour and the level of physical activity, are directly related to both obesity [21] and depression [22] and could, therefore, underlie the relationship between the two. As alcohol consumption, smoking behaviour and levels of physical activity are different between ethnic groups [23–25] they could be ethnicity-differential contributing factors explaining the obesity-depression relationship. Moreover, certain diseases associated with obesity, such as diabetes and heart disease, which have been shown to influence depressive symptoms [26], are also more prevalent among certain ethnic groups [27].

We investigated the relationship between obesity and depressed mood and whether this association was different among six ethnic groups living in Amsterdam, a European city. Ethnic differences will be explored in different models with adjustment for sociodemographic factors, health behaviours and somatic health to explore the effect these variables have on the obesity/depression association among ethnic groups.

## Methods

### Study population

The HEalthy LIfe in an Urban Setting (HELIUS) study is a multi-ethnic cohort study conducted in Amsterdam, the Netherlands which has been described in detail elsewhere [28]. In brief, baseline data collection took place in 2011–2015 and included people aged 18–70 years from different ethnic origins. Participants of Dutch, Surinamese, Turkish, Moroccan and Ghanaian ethnic origin were randomly selected, by ethnic group, from the municipal register ensuring roughly equal numbers from each ethnic group. Socio-historical information on the ethnic minority groups included in this study can be found elsewhere [29]. Data were collected by questionnaire and a physical examination in which biological samples were also obtained. Participants unable to fill out questionnaires in Dutch were offered questionnaires in English and Turkish, or assistance from an ethnically matched, trained interviewer (all ethnic minority groups).

For the current study, cross-sectional baseline data were used, including 22,165 participants for whom questionnaire data as well as data from the physical examination were available. We excluded those of Javanese Surinamese (*n* = 233) or unknown Surinamese (*n* = 267) origin due to small numbers. We also excluded those with another/unknown ethnic origin (*n* = 48). Additionally, due to low numbers, those who were underweight (BMI < 18.5 kg/m^2^, *n* = 354), as well as those with missing BMI data (*n* = 22) or missing depression scores (*n* = 211) were also excluded, which resulted in an analytic sample of 21,030 participants (4477 Dutch, 2938 South-Asian Surinamese, 4035 African Surinamese, 2265 Ghanaian, 3520 Turkish, 3795 Moroccan origin).

### Ethnicity

Ethnicity was defined according to the country of birth of the participant as well as that of his/her parents, which is currently the most widely accepted assessment of ethnicity in the Netherlands [30]. Specifically, a participant is considered as of non-Dutch ethnic origin if he/she fulfils either of the following criteria: (1) he or she was born abroad and has at least one parent born abroad (first generation); or (2) he or she was born in the Netherlands but both his/her parents were born abroad. There were no third generation ethnic minority individuals in the cohort. Of the Surinamese immigrants in the Netherlands, approximately 80% are either African or South-Asian origin. Surinamese subgroups were classified according to self-reported ethnic origin. Participants were considered as of Dutch origin if the both parents were born in the Netherlands.

### Depressed mood

Depressed mood was assessed using the Patient Health Questionnaire-9 (PHQ-9), an instrument consisting of 9 items with 4 response options for each item [never (0), several days (1), more than half the days (2) and nearly every day (3)], giving a sum score range of 0–27, with higher scores indicating more depressive symptoms [31]. If one of the items was missing (*n* = 406, 1.7%), the mean score of the other eight items was used to replace the missing item. If more than one item was missing, the variable was considered missing. This questionnaire assesses depressive symptoms during the previous 2 weeks. Depressed mood was considered present when a person had a PHQ-9 score equal or greater than 10. This cut-off has a sensitivity and specificity of 88% of predicting major depressive disorder [31], is a commonly used cut-off [32], and has been shown to be consistent across American ethnic groups [33]. The PHQ-9 has been shown to measure the same concepts across all six ethnic groups included in this study, and there are no systematic differences in reporting depressive symptoms between the groups [34].

### Anthropometric measurements

Obesity measures used were BMI and waist circumference, a measure of abdominal visceral fat, which is generally seen as a more pathogenic metabolic risk factor [35]. Body weight and body height were measured in duplicate by a trained research assistant in barefoot participants wearing light clothes only. Waist circumference was measured in duplicate using a tape measure at the level midway between the lowest rib margin and the iliac crest. BMI was calculated as weight in kilograms divided by height squared in meters (kg/m^2^). In addition to a continuous indicator, BMI categories were made according to the World Health Organization classification, 18.5 to < 25 kg/m^2^ (normal), 25 to < 30 kg/m^2^ (overweight), ≥ 30 kg/m^2^ (obese). Waist circumference (cm), continuous and as sex-specific quartiles, were used as a measure of abdominal obesity. As the original units were small and to make the results of the two continuous obesity measures more comparable, both values were standardised.

### Covariates

Based on previous literature, adjustments were made for three different groups of covariates [22, 26, 36, 37]. Socio-demographic covariates included age, gender and level of education (the highest level of education completed with a diploma or certificate of proficiency in the Netherlands or in the country of origin). The second group of covariates were health behaviours which included physical activity (achieving the Dutch guideline for physical activity) measured with the SQUASH questionnaire [38], smoking (current, former, never) and alcohol use. Alcohol intake was reported as weekly or monthly frequency and typical number of consumptions per drinking day. This was subsequently converted into 3 categories non-drinker, low-moderate (0–14 drinks/week women, 0–21 drinks men), heavy (≥ 14 drinks/week women, ≥ 21 drinks/week men) assuming mode values for each frequency category. The third group of covariates was somatic health. Somatic health was derived from the number of self-reported diseases: presence of hypertension, diabetes, cardiovascular diseases (cardiovascular disease, cardiovascular accident, myocardial infarction), chronic lung disorders (asthma, chronic bronchitis, lung emphysema), osteoarthritis (arthrosis, rheumatoid arthritis) and cancer. Participants were asked if they had been diagnosed by a doctor with any of these diseases. Data were complete for all covariates with the exception of physical activity (26 missing) and waist circumference (16 missing) thus missing data was ignored.

### Statistical analysis

Baseline socio-demographic characteristics, health behaviours, somatic and anthropometric data are presented according to ethnic origin. Initially the overall association between obesity measures (both continuous and categorical) and depressed mood was analysed adjusted for socio-demographic variables (age, gender, educational level). Normal BMI and waist circumference in the lowest quartile were used as reference categories. The analysis estimating the association of waist circumference with depressed mood also included adjustment for height (cm) to adjust for body size.

To examine ethnic differences in the association between obesity measures and depressed mood, interaction terms for the product of the dichotomised obesity measures [obese/waist circumference highest quartile (yes/no)] with ethnicity were added to the regression models. Three models of increasing complexity were made: the first model was adjusted for socio-demographics and ethnicity (model 1), the second added health behaviours (model 2) and the third added somatic health (model 3). The Dutch group was taken as a reference group, although we also ran models varying the reference groups to test for differences between all ethnic groups. Results of significant modifications of the obesity-depressed mood relationship by ethnicity, as identified by the overall interaction term, were stratified and the odds ratios for the individual ethnic groups were simultaneously displayed in a figure along with the overall odds ratio. Three separate models were made to illustrate whether the ethnic differences were present after adjustment for socio-demographic variables (model 1), after additional adjustment for health behaviours (model 2), and after additional adjustment for number of chronic diseases (model 3). We also performed a couple of post hoc analyses. Given that some studies have found that ethnic differences in the obesity-mood relationship are gender specific, we also tested for gender*ethnicity*obesity measures interaction. Additionally, we also examined whether length of residence modified the obesity-depression relationship of the non-Dutch residence by adding a obesity*length_of_stay variable to the models. Statistical significance was set at *P* < 0.05 (*P* < 0.1 for the testing of interaction terms) and all analyses were performed in SPSS version 22 (Inc., Chicago, Illinois, USA).

## Results

Table 1 shows that the Dutch and African Surinamese population were moderate drinkers whilst the other ethnic groups were mostly non-drinkers. Smoking was particularly low among Ghanaians. Another notable difference was that depressed mood was more prevalent in all ethnic minorities compared to those of Dutch origin, with particularly high prevalence in people of Turkish, Moroccan and South-Asian Surinamese origin. The prevalence of overweight and particularly obesity was higher among all ethnic minority groups as compared with Dutch, with the highest obesity rates among those of Ghanaian and Turkish origin.

**Table 1:** Characteristics of the HELIUS study participants by ethnicity

Variables	Dutch (*n* = 4477)	South Asian Surinamese (*n* = 2938)	African Surinamese (*n* = 4035)	Ghanaian (*n* = 2265)	Turkish (*n* = 3520)	Moroccan (*n* = 3795)
Sex [*n* (%)], male	2074 (46)	1335 (45)	1567 (39)	880 (39)	1593 (45)	1482 (39)
Age (years), mean (SD)	46.3 (13.9)	45.8 (13.3)	48.1 (12.4)	44.7 (11.1)	40.5 (12.1)	40.6 (12.9)
1st generation (%)	4477 (100%)	2270 (77)	8380 (88)	2164 (96)	2481 (71)	2624 (69)
Residence duration (years)	N/A	33.1 (8.7)	32.1 (10.5)	18.3 (8.1)	28.8 (8.2)	29.0 (8.9)
Educational level						
No or elementary [*n* (%)]	146 (3)	419 (14)	223 (6)	643 (28)	1183 (31)	1103 (31)
Lower vocational or lower secondary [*n* (%)]	636 (14)	990 (34)	1437 (36)	923 (40)	894(25)	693 (19)
Intermediate vocational /higher/secondary [*n* (%)]	997 (23)	857 (29)	1449 (35)	561 (26)	997 (29)	1262 (33)
Higher vocational or university [*n* (%)]	2698 (60)	672 (23)	926 (23)	138 (6)	526 (15)	675 (17)
Smoking status						
Never [*n* (%)]	1640 (37)	1699 (58)	1966 (49)	1974 (87)	1655 (47)	2802 (74)
Former [*n* (%)]	1731 (38)	417 (14)	808 (19)	193 (8)	660 (18)	489 (13)
Current [*n* (%)]	1106 (25)	822 (28)	1261 (32)	98 (4)	1205 (34)	504 (13)
Alcohol						
Non-drinker [*n* (%)]	397 (9)	1248 (43)	1248 (31)	1179 (52)	2708 (77)	3508 (92)
Low-moderate [*n* (%)]	3637 (81)	1577(54)	2675(66)	1065(47)	775(22)	266(7)
Heavy [*n* (%)]	443 (10)	81 (3)	112 (3)	21 (1)	37 (1)	21 (1)
Physical activity [*n* (% achieving guideline)]^a,b^	3383 (76)	1572 (53)	2484 (61)	1212 (53)	1483 (42)	1786 (47)
Chronic diseases [*n* (% with *n* > 1)]	291 (6.5)	599 (20.4)	691 (17.1)	277 (12.2)	468 (13.3)	374 (9.9)
Chronic diseases, median (IQR)	0(0–0)	0 (0–1)	0 (0–1)	0 (0–1)	0 (0–1)	0 (0–1)
Height (cm), mean (SD)	175.5 (9.5)	164.5 (9.3)	168.6 (4.6)	165.5 (7.8)	165.3 (9.4)	166.4 (9.2)
BMI (kg/m^2^), mean (SD)	24.8 (4.2)	26.6 (4.6)	28.0 (5.4)	28.6 (5.0)	28.7 (5.6)	27.7 (5.1)
BMI categories						
Normal (18.5 to < 25 kg/m^2^) [*n* (%)]	2657 (59)	1231 (42)	1308 (33)	557 (25)	949 (27)	1242 (33)
Overweight (25 to < 30 kg/m^2^) [*n* (%)]	1359 (31)	1135 (39)	1506 (37)	930 (41)	1327 (38)	1435 (38)
Obese (≥ 30 kg/m^2^) [*n* (%)]	461 (10)	572 (19)	1221 (30)	778 (34)	1244 (35)	1118 (29)
Waist circumference (cm)						
Mean (SD)	89.2 (11.9)	91.2 (11.6)	92.4 (12.7)	91.9 (11.6)	93.9 (13.4)	92.8 (13.2)
Quartile 1^c^	1461 (32)	642 (22)	921 (23)	444 (20)	705 (20)	818 (22)
Quartile 2	1274 (29)	805 (27)	1010 (25)	565 (25)	797 (23)	881 (23)
Quartile 3	935 (21)	838 (29)	1003 (25)	657 (29)	916 (26)	1008 (26)
Quartile 4	805 (18)	651 (22)	1095 (27)	597 (26)	1099 (31)	1087 (29)
Depressed mood score (PHQ-9) median (IQR)	3.0 (1.0–5.0)	4.0 (1.0–8.0)	3.0 (0.0–5.0)	2.0 (0.0–5.0)	5.0 (2.0–9.0)	4.0 (2.0–8.0)
Depressed mood, yes [*n* (%)]^2^	316 (7)	548 (19)	429 (11)	209 (9)	820 (23)	793 (21)

*SD* standard deviation, *BMI* body mass index, *IQR* interquartile range

Low = less than 1 drink a week, moderate = men: 1–21 drinks/week; women: 1–14/week, heavy = men: >21/week; women: >14/week

^a^Achieved the Dutch national guidelines of ≥ 5 days a week 30 mins moderate-intensive activity

^b ^PHQ-9 sumscore ≥10

^c^Quartile 1 (<86.0cm)^m^ (<80.1cm)^f^, Quartile 2 (86.0–<93.4cm)^m^(80.1–<89.65cm)^f^, Quartile 3 (93.4–<101.5cm)^m^(89.7–<100.3cm)^f^, Quartile 4 (>101.5cm)^m^(>100.3cm)^f^; *m* males, f females

Logistic regression analysis showed that both higher BMI and higher waist circumference were significantly associated with depressed mood among the total sample, after adjustment for age, sex and educational level [Odds Ratio (OR) for 1 SD higher BMI = 1.16; 95% confidence interval (CI) 1.12–1.21 and for 1 SD higher waist circumference: OR = 1.20; 95% CI 1.15–1.25] (supplementary Table 1). Dividing the obesity measures into categories showed that it was participants in the highest categories that had the greatest odds of depressed mood. Thus, participants with obesity or having a waist circumference in the highest quartile had greater odds of having depressed mood than those not in the extreme weight categories (OR = 1.43; 95% CI 1.29–1.54, OR = 1.56; 95% CI 1.38–1.77, respectively).

The relationship between obesity measures and depressed mood was significantly different across ethnic groups, as evidenced by significant overall p-values for the interaction terms ethnicity *obesity measures (supplementary Table 2), suggesting that the association between obesity measures and depressed mood is not consistent across ethnic groups. These interactions were significant for both obesity and waist circumference in the model adjusted for socio-demographic factors where the p values for the overall interaction terms were 0.03 and 0.05 for obesity and a high waist circumference, respectively. The effect modification of ethnicity on obesity measures remained significant with the addition of health behaviours [*p* value = 0.07 (obesity) 0.04 (waist circumference)] and persisted after the addition of somatic health for having a high waist circumference (*p* value = 0.06). However, the significance was diminished for the interaction between obesity*ethnicity after adjustment for somatic health (*p* value = 0.21).

**Fig. 1:**
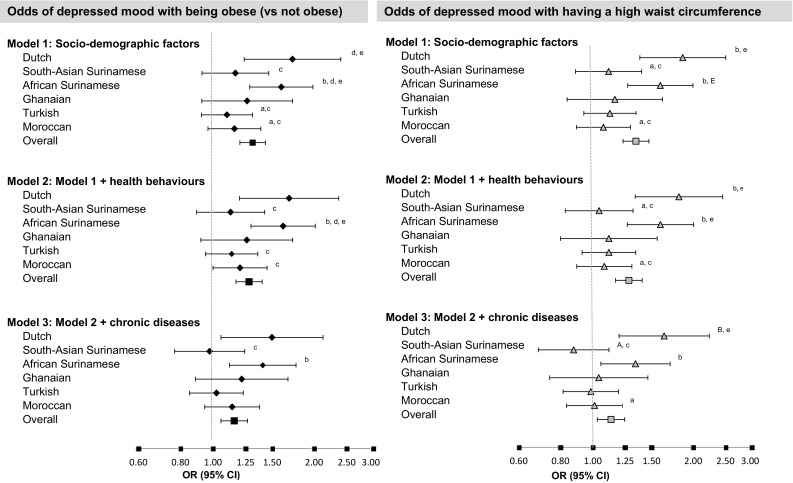
The association between obesity (diamonds) and waist circumference (triangles) with depressive symptoms (PHQ-9 score ≥ 10) by ethnicity, adjusted for covariates in different models. ^a^Significantly different (*P* < 0.05) from Dutch, ^b^South-Asian Surinamese, ^c^African Surinamese, ^d^Turkish, ^e^Moroccan. ^A^Significantly different (*P* < 0.01) from Dutch, ^B^South-Asian Surinamese, ^E^Moroccan. Models ^1^:Socio-demographic: age, sex and education, ^2^:Socio-demographic plus lifestyle: smoking, alcohol use and physical activity, ^3^:Socio-demographic

To illustrate the ethnicity interaction, Fig. 1 shows the odds ratios for the relationship between obesity measures and depressed mood, stratified by ethnicity. Model 1 showed that Dutch and African Surinamese had a significant, positive association between obesity and depressed mood (OR = 1.72; 95% CI 1.24–2.40, OR = 1.60; 95% CI 1.29–1.98, respectively), whilst obesity in South-Asian Surinamese, Ghanaian, Moroccans and Turks showed a weaker non-significant association with depressed mood. Waist circumference quartiles showed a similar pattern with slightly larger differences between the ethnic groups. For those of Dutch and African Surinamese origins a high waist circumference had a positive association with depressed mood (OR = 1.86; 95% CI 1.38–2.50, OR = 1.59; 95% CI 1.27–2.00, respectively) whilst in South-Asian Surinamese, Ghanaian, Moroccans and Turks having a high waist circumference had a weaker non-significant association with depressed mood. This pattern of associations between ethnic groups remained the same after adjustment for health behaviour and somatic health and if anything, were slightly more pronounce for having a waist circumference in the highest quartile after adjustment for both health behaviours and somatic health.

Three way interaction terms showed that no gender*ethnic differences were present in the obesity–depression relationship [for both obese vs non-obese and waist circumference in quartile 4 (y/n)], both before and after adjustment for health behaviours and somatic health. Additionally, the length of residence in Amsterdam did not modify the relationship between obesity and depression.

## Discussion

This study aimed to investigate whether the cross-sectional relationship between BMI or waist circumference (continuous and in categories) and the presence of depressed mood is consistent across six ethnic groups, i.e. Dutch, South-Asian Surinamese, African Surinamese, Turkish, Moroccan and Ghanaian, living in the same European city (Amsterdam, The Netherlands). Our results indicate that the association between being obese (BMI ≥ 30 kg/m^2^) and depressed mood tend to differ across ethnic groups: this association was stronger and statistically significant among Dutch and African Surinamese but weaker among those of Ghanaian, South-Asian Surinamese, Turkish or Moroccan origin. This pattern remained after additional adjustment for health behaviours and somatic health, illustrating that these factors are not largely involved in explaining the ethnic differences in the association between obesity and depressed mood.

Our study is one of the first to compare the relationship of BMI/waist circumference with depressed mood among different ethnic groups in Europe. The finding that obesity measures do not have a unanimous association with depressed mood across ethnic groups has been found in some other studies in the US [11–17]. Our results are partially in accordance with the previously performed American studies in that these studies also found that obesity increased the risk of depressed mood in the (non-Hispanic) White population but not the other ethnic groups [Mexican, Hispanic or Black (African American)] [11, 13, 15]. However, in our study, we found that African Surinamese with obesity were also relatively more likely to suffer from depressed mood.

Not all earlier studies found that ethnicity modifies the relationship between BMI/obesity and depressed mood. Remigio-Baker et al. [19] examined the cross-sectional relationship between visceral fat measured by computed tomography and elevated depressed symptoms in 1017 men and women aged 47–84. They found no significant interaction between elevated depressive symptoms and race/ethnicity (White, Black, Chinese and Hispanic) for visceral fat; however, they attributed this to lack of statistical power. Another study by Dong et al. [18] found that extreme obesity was consistently associated with an increased risk for depression across two racial groups (African American vs. European American). The population of this study differed considerably from ours due to the large proportion of extremely obese (BMI > 40 kg/m^2^) individuals (44% across racial groups), whilst only 2.2% of our participants had a BMI > 40 kg/m^2^.

Considering that the observed ethnic differences in the association between obesity and depression remained after adjustment for health behaviours and, in the case of central adiposity, somatic health, it is unlikely that these are strong mechanisms. Additional explanations for the differences across ethnic groups could be based on sociocultural differences. Higher prevalence of obesity among certain ethnic groups may lead to protective social norms. Among cultures where a “larger body size” is the norm, obesity is socially more acceptable [39] resulting in less body dissatisfaction, less mental stress [40, 41] and a buffering from weight discrimination. All of these can lead to a higher self-esteem which itself is a protective factor for depressed mood [42–44]. In America, perceived weight discrimination has been found to be greater in White Americans, where the relationship between obesity and depression is stronger than in African Americans [45]. Among our population sample, obesity was most prevalent among the Turkish, Moroccan and Ghanaian populations in whom, along with the South-Asian Surinamese, the association between obesity and depression is weakest. However, a study of Turkish and Moroccan residence of Amsterdam has shown that this population does not express a preference for a larger body size [46]. Hence, body satisfaction is unlikely to explain the weak association between obesity and depression for the Turkish and Moroccan population, although social acceptance of a larger body size may be a contributing factor, especially considering that a large proportion of the overweight Turkish and Moroccan men perceived their weight to be average. A study by Hoeninka et al. in the HELIUS population found that African Surinamese and Ghanaians experienced body dissatisfaction at a higher BMI than did Dutch origin populations [47]. Hence, for the Dutch population, this is consistent with our hypothesis that body dissatisfaction and, therefore, also depression, is greater in ethnic groups where the prevalence of obesity is lower. However, this does not explain the strong obesity-depression relationship among African Surinamese, thus this theory does not hold for this group. Another possibility is that how strongly being obese is related to depression may be dependent on how long the non-Dutch participants have resided in the Netherlands. The assumption being that the longer the duration of residence, the more assimilated a person is to the Dutch culture and, therefore, the more similar they would be in terms of their relationship between their obesity status and depression. However, given that the length of residence is similar between all ethnic groups (except for the Ghanaians), and in particular the fact that both Surinamese ethnic groups migrated at the same time, this could not explain the differences between groups. Furthermore, there was no interaction between length of stay and obesity. Alternatively, in addition to ethnic normative differences, another explanation for ethnic differences in the obesity-depression association could be an ethnic-differential pathophysiology. Several biological mechanisms, ranging from systemic inflammation, leptin resistance, metabolic syndrome disturbances, dysregulated hypothalamus–pituitary–adrenal axis (HPA-axis) have been proposed to be linking mechanisms between depression and obesity as these all occur in both conditions [48]. There are indications that occurrence of these biological mechanisms are ethnicity dependent [49]. We should also take into account the fact that the relationship between obesity and depression is bidirectional [8] and, therefore, it could be that when suffering from a depressed mood some ethnic groups gain weight whilst other remain the same or perhaps even loose weight.

This study is the first to examine the association of obesity with depressed mood among a multi-ethnic European sample. The strength of this study is its large sample size comprising of roughly equal numbers of different ethnic groups, including a variety of migrant groups found in Europe along with a large number of covariates. Additionally, we had multiple measures of obesity. The inclusion of waist circumference is important as waist circumference is generally considered a more pathogenic metabolic risk factor.

There are a few limitations. First, this study was cross-sectional and as such no causal pathways could be investigated. However, longitudinal studies have indicated that having obesity is a risk factor for developing depression but it is generally accepted that the relationship is bidirectional [8]. Second, the PHQ-9 is not equivalent to a diagnosis of clinical depression and only captures depressed mood, thus these results are not generalizable to those with clinical depression. Finally, adjustment for health behaviours excluded nutritional intake, and the measurement of physical activity was subjective, therefore, leaving potential for residual confounding.

We found that obesity measures were positively associated with depressed mood but only in certain ethnic groups. The association was stronger and statistically significant in the Dutch and African Surinamese whereas the association was weaker and not statistically significant in South-Asian Surinamese, Ghanaian, Moroccan and Turkish origin groups. The pattern among ethnic groups remained the same even after adjusting for differences in health behaviours and somatic health and became even more pronounced for high waist circumference compared to having obesity. Knowing that the relationship between obesity and depressed mood is not universal among ethnic groups, may help target prevention strategies with the knowledge that for some ethnic groups, programmes aimed at targeting obesity may result in an improvement in both somatic and mental health and whilst in other groups the improvement in physical health would be the main focus. Future studies should explore whether differential social-cultural based normative values or underlying pathophysiology across ethnic groups explain why obesity and depression are strongly related in some but not all ethnic groups.

## Electronic supplementary material

Below is the link to the electronic supplementary material.

Supplementary material 1 (DOCX 20 KB)
