# Supplementary material for: The relation between obesity and depressed mood in a multi-ethnic population. The HELIUS study
Source: Soc Psychiatry Psychiatr Epidemiol. 2018 Apr 11;53(6):629–38. doi: 10.1007/s00127-018-1512-3 (PMC5959973; doi:10.1007/s00127-018-1512-3)
Supplement: Supplementary file 1 — Supplementary material 1 (DOCX 20 KB) [file 127_2018_1512_MOESM1_ESM.docx]

| **Supplementary Table 1**: The association of BMI and waist circumference with depressed mood (PHQ-9 score >=10) (N=21,030) | | | |
| --- | --- | --- | --- |
|  | Socio-demographic adjusted^a^ | | |
|  | OR | 95% CI | P-value |
| Body mass index (per SD increase) | **1.16** | **1.12-1.21** | **<0.001** |
| Body mass index categories |  |  |  |
| Normal (18.5 – 24.9 kg/m^2^) | 1.00 | (Reference) |  |
| Overweight (25 – 29.9 kg/m^2^) | **1.16** | **(1.05-1.27)** | **0.04** |
| Obese (≥30 kg/m^2^) | **1.43** | **(1.29-1.54)** | **<0.001** |
| Waist Circumference (per SD increase) | **1.20** | **(1.15-1.25)** | **<0.001** |
| Waist Circumference categories |  |  |  |
| Quartile 1 (<86.0cm)^m^ (<80.1cm)^f^ | 1.00 | (Reference) |  |
| Quartile 2 (86.0-<93.4cm)^m^(80.1-<89.65cm)^f^ | 1.11 | (0.98-1.25) | 0.97 |
| Quartile 3 (93.4-<101.5cm)^m^(89.7-<100.3cm)^f^ | **1.29** | **(1.14-1.45)** | **<0.001** |
| Quartile 4 (>101.5cm)^m^(>100.3cm)^f^ | **1.56** | **(1.38-1.77)** | **<0.001** |
| OR=odds ratio  ^a^Adjusted for age, gender, educational level (and body height in the case of waist circumference)  ^m^ Males ^f^ Females | | | |

| **Supplementary Table 2** Interaction terms showing which ethnic groups significantly differ from the Dutch in the relationship between obesity measures and depressed mood (y/n) (n=21,030) | | | | | | | | | | | | | | | | | | | |
| --- | --- | --- | --- | --- | --- | --- | --- | --- | --- | --- | --- | --- | --- | --- | --- | --- | --- | --- | --- |
|  |  | Model 1:  Socio-demographic adjusted | | | |  | | Model 2:  Model 1 + health behaviours | | | | |  | | Model 3:  Model 2 + chronic diseases | | | | |
|  |  | OR | | 95% CI | P-value | | OR | | | 95% CI | | P-value | | OR | | | 95% CI | | P-value |
| Categorical BMI | |  | | | | |  | | | | | | |  | | | | | |
| Ethnicity * obesity | |  | | | **0.03** | |  | | | | **0.07** | | |  | | | | 0.21 | |
| South Asian Surinamese * obesity (vs non obese) | | 0.72 | (0.49-1.05) | | **0.09** | | 0.73 | | (0.49-1.07) | | 0.11 | | | 0.70 | | (0.47-1.04) | | 0.08 | |
| African Surinamese * obesity (vs non obese) | | 0.97 | (0.67-1.42) | | 0.89 | | 0.99 | | (0.67-1.45) | | 0.90 | | | 0.96 | | (0.64-1.38) | | 0.83 | |
| Ghanaian * obesity (vs non obese) | | 0.73 | (0.48-1.12) | | 0.15 | | 0.72 | | (0.46-1.10) | | 0.11 | | | 0.77 | | (0.50-1.17) | | 0.24 | |
| Turkish * obesity (vs non obese) | | 0.70 | (0.49-0.99) | | **0.05** | | 0.74 | | (0.51-1.06) | | **0.08** | | | 0.75 | | (0.52-1.07) | | 0.12 | |
| Moroccan * obesity (vs non obese) | | 0.66 | (0.46-0.94) | | **0.02** | | 0.71 | | (0.50-1.02) | | **0.05** | | | 0.75 | | (0.52-1.08) | | 0.13 | |
| Categorical Waist circumference | |  | | | | |  | | | | | | |  | | | | | |
| Ethnicity * Q4 | |  | | | **0.05** | |  | | | | **0.04** | | |  | | | | **0.06** | |
| South Asian Surinamese * Q4 (vs non Q4) | | 0.70 | (0.50-0.99) | | **0.05** | | 0.68 | | (0.48-0.97) | | **0.03** | | | 0.62 | | (0.43-0.88) | | **0.01** | |
| African Surinamese * Q4 (vs non Q4) | | 0.95 | (0.68-1.34) | | 0.79 | | 0.96 | | (0.68-1.36) | | 0.83 | | | 0.89 | | (0.63-1.27) | | 0.53 | |
| Ghanaian * Q4 (vs non Q4) | | 0.71 | (0.47-1.07) | | 0.11 | | 0.69 | | (0.45-1.04) | | **0.07** | | | 0.70 | | (0.46-1.06) | | 0.10 | |
| Turkish * Q4 (vs non Q4) | | 0.75 | (0.55-1.03) | | **0.08** | | 0.77 | | (0.56-1.06) | | **0.10** | | | 0.76 | | (0.55-1.04) | | **0.03** | |
| Moroccan * Q4 (vs non Q4) | | 0.66 | (0.48-0.91) | | **0.01** | | 0.68 | | (0.49-0.94) | | **0.02** | | | 0.69 | | (0.50-0.96) | | **0.03** | |
| Model 1: Adjusted for age, gender, educational level, and ethnicity (and height in the case of waist circumference)  Model 2: As for model 1 plus smoking, alcohol use and achieving norm for physical activity  Model 3: As for model 2 plus number of chronic diseases | | | | | | | | | | | | | | | | | | | |
